# *Listeria monocytogenes* Infection in Hairy Cell Leukemia: A Case Report and Literature Review

**DOI:** 10.1155/2018/5616898

**Published:** 2018-01-30

**Authors:** James C. Barton, Hayward S. Edmunds

**Affiliations:** ^1^Department of Medicine, University of Alabama at Birmingham, Birmingham, AL, USA; ^2^Southern Iron Disorders Center, Birmingham, AL, USA; ^3^Department of Medicine, Brookwood Medical Center, Birmingham, AL, USA; ^4^Cunningham Pathology Associates, Birmingham, AL, USA

## Abstract

*Listeria monocytogenes* infections have been described in patients with diverse types of malignancy, especially leukemia. We report the case of a 65-year-old man with previously untreated hairy cell leukemia characterized by CD5 positivity and trisomy 12 (3% of blood lymphocytes) who developed bacteremia due to *L. monocytogenes* serotype 1/2b. We summarize clinical features and treatment of this patient and five previously reported patients with hairy cell leukemia who also had *L. monocytogenes* infections. All six patients were men. Their mean age at infection diagnosis was 70 y. Three men had undergone splenectomy 4–11 y before they developed *L. monocytogenes* infection. The central nervous system was the primary site of infection in four men. Bacteremia alone occurred in two other men. At diagnosis of infection, one man was receiving antileukemia chemotherapy and another man was receiving treatment for Kaposi's sarcoma. Two other patients had other comorbid conditions. All six men recovered from their infections.

## 1. Introduction

Hairy cell leukemia (HCL) is characterized by small mature B-lymphocytes with distinctive morphology and surface protein expression, hemocytopenias (especially neutropenia and monocytopenia), marrow fibrosis, splenomegaly, and an indolent clinical course [1]. The median age of patients with HCL is between 50 and 55 y [1]. Approximately 80% of persons with HCL are men [1]. Some persons with HCL develop autoimmune conditions unrelated to the severity of HCL [1, 2]. Cladribine therapy induces durable hematologic remissions in a high proportion of previously untreated patients [3].

We report the case of a man with previously untreated HCL who developed *Listeria monocytogenes* bacteremia. We summarize clinical features and treatment of this patient and five previously reported patients with HCL who were also diagnosed to have *L. monocytogenes* infections and discuss manifestations, management, and outcomes of *L. monocytogenes* infections in persons with HCL.

## 2. Case Report

A 64-year-old white man presented with a 10-month history of fatigue, lymphocytosis, microcytosis (elevated RDW), and subnormal platelet counts. He had bipolar disorder. Five months before presentation, he was treated as an outpatient for pneumonia not otherwise specified. Daily medications were duloxetine, lamotrigine, olanzapine, acetaminophen, fish oil, and melatonin. He tended horses and handled their silage daily. Physical examination revealed mild pallor. Lymph nodes, liver, and spleen were not enlarged.

**Figure 1:**
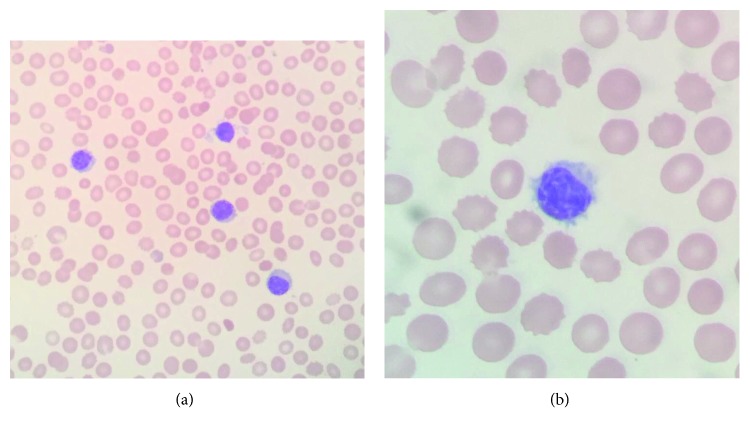
Photomicrographs of blood leukemic B-lymphocytes in a man with untreated hairy cell leukemia. (a) Original magnification 400x. (b) Original magnification 1000x.

**Figure 2:**
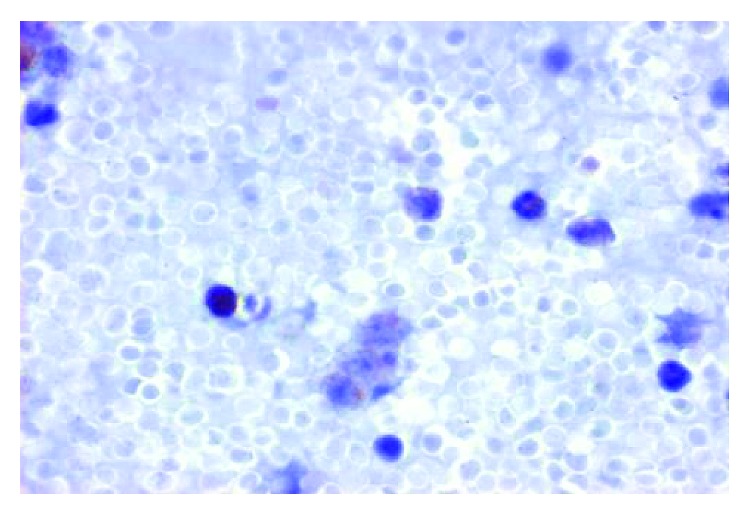
Photomicrograph of blood leukemic B-lymphocytes in a man with treated hairy cell leukemia stained to demonstrate tartrate-resistant acid phosphatase. Rare lymphoid cells are weakly to moderately positive (Genoptix, Carlsbad, CA) (original magnification 400x).

Complete blood count revealed hemoglobin 110 g/L, erythrocytes 4.79 × 10^12^/L, mean corpuscular volume 73 fL, red blood cell distribution width 17.2%, leukocytes 15.6 × 10^9^/L, neutrophils 4.4 × 10^9^/L, lymphocytes 10.5 × 10^9^/L, monocytes 0.5 × 10^9^/L, and platelets 125 × 10^9^/L. Serum iron level was 5.7 *µ*mol/L, transferrin saturation was 7%, and serum ferritin was 23 pmol/L. Comprehensive chemistry profile values were within respective reference limits. Total immunoglobulin (Ig) G was 9.76 g/L. IgG subclass values were within respective reference limits. A HCL immunophenotype was detected in 68% of blood lymphocytes by flow cytometry (Table 1). Morphology of his leukemic blood lymphocytes is displayed in Figures 1 and 2. Fluorescent in situ hybridization revealed trisomy 12 in 3% of blood lymphocytes (reference < 1.3%). t(11;14) was not detected. He declined to undergo bone marrow evaluation or CT scanning.

Fecal occult blood testing was positive, although upper gastrointestinal tract endoscopy and colonoscopy did not reveal a site of blood loss. He was treated with four 500 mg infusions of iron as iron dextran. His fatigue resolved and his hemoglobin, erythrocytes, and mean corpuscular volume increased to 133 g/L, 4.85 × 10^12^/L, and 88 fL, respectively. Transferrin saturation and serum ferritin increased to 16% and 357 pmol/L, respectively.

Fifteen months after diagnosis, his physical examination revealed mild pallor. Hemoglobin was 102 g/L, neutrophil count was 6.2 × 10^9^/L, midrange cells were 5.8 × 10^9^/L, lymphocyte count was 36.9 × 10^9^/L, and platelet count was 72 × 10^9^/L. There was no evidence of recurrent iron deficiency.

At age 65 y, 16 months after diagnosis, he was admitted to hospital because he had temperature 103.1° and chills. Hemoglobin was 99 g/L, erythrocytes were 3.10 × 10^12^/L, mean corpuscular volume was 101 fL, leukocytes were 59.9 × 10^9^/L, neutrophils were 3.0 × 10^9^/L, lymphocytes were 56.9 × 10^9^/L (88% with HCL immunophenotypes), and platelets were 83 × 10^9^/L. The immunophenotype of his monoclonal blood lymphocytes was unchanged since diagnosis (Table 1). There was no apparent source of infection. He was treated initially with oral acetaminophen and intravenous fluids. On the second hospital day, he was treated empirically with oral levofloxacin 750 mg daily and became afebrile. He was discharged on the fourth hospital day to continue taking levofloxacin 750 mg daily (7 days total). He recovered fully. After discharge, it was reported that aerobic and anaerobic blood cultures drawn at hospital admission revealed a Gram-positive rod interpreted as *Corynebacterium* sp. Further study revealed that the isolate was *L. monocytogenes*. Neither the patient nor his wife had consumed uncooked or unpasteurized food items sometimes associated with *Listeria* transmission.

Subtyping of the present *L. monocytogenes* isolate was performed at the National Enteric Reference Laboratory (Centers for Disease Control and Prevention, Atlanta, GA) using growth phenotype on Trypticase™ Soy Agar with 5% Sheep Blood (BD Diagnostics, Sparks, MD) and nucleic acid-based analyses (average nucleotide identity and AccuProbe^®^ culture identification testing (Hologic Inc., Marlborough, MA)). Testing detected serotype 1/2b.

In month 18 after diagnosis (Table 1), he was treated with subcutaneous cladribine therapy [1, 4] because his recurrent anemia was best explained by decreased erythropoiesis due to marrow progression of HCL [1]. In month 23 (4 months after last cladribine injection), his hemoglobin was 118 g/L, erythrocytes were 4.09 × 10^12^/L, mean corpuscular volume was 97 fL, leukocytes were 3.6 × 10^9^/L, neutrophils were 2.5 × 10^9^/L, lymphocytes were 0.8 × 10^9^/L, and platelets were 113 × 10^9^/L. Three percent of blood lymphocytes had a HCL immunophenotype (Table 1).

Mutation analysis to detect *BRAF* p.V600E (c.1799T > A) was performed for the first time in month 28 after diagnosis and 10 months after cladribine therapy (Table 1) by Genoptix (Carlsbad, CA) using the Cobas^®^ 4800 V600 Mutation Test (Roche Molecular Systems, Inc., Branchburg, NJ). *BRAF* p.V600E was not detected.

## 3. Literature Search

We performed a computerized search of the National Library of Medicine and other sources to identify reports of patients with HCL who developed *L. monocytogenes* infections. We enhanced the computerized search by reviewing details of individual patients in selected case series of *L. monocytogenes* infections.

## 4. Six Patients with Hairy Cell Leukemia and *L. monocytogenes* Infections

We identified previous reports of *L. monocytogenes* infections in five patients with HCL, although their HCL immunophenotypes were not reported [5–9] (Table 2). Including the present patient, all six were men. Mean age of the six men at infection diagnosis was 70 y (standard deviation 13 y). Three men had undergone splenectomy 4–11 y before they developed *L. monocytogenes* infection. The central nervous system was the primary site of infection in four men. Bacteremia alone occurred in two other men. Possible sources of *L. monocytogenes* were reported in two men. At diagnosis of infection, one man was receiving antileukemia chemotherapy and another man was receiving treatment for Kaposi's sarcoma. Two other patients also had other comorbid conditions. Antibiotic therapy was reported in five men, four of whom were treated with ampicillin in combination with other agents. All six men recovered from their infections (Table 2). *L. monocytogenes* serotyping and detailed flow cytometry and fluorescent in situ hybridization analyses were not described in the previous five reports [5–9].

## 5. Discussion

*L. monocytogenes* infection in patients with HCL has been reported infrequently [5–9]. The mean age and sex of the five previously reported cases and the present patient were typical of patients with HCL [1]. Splenectomy, current chemotherapy, and other comorbid conditions were common among these six patients. Four men had infections of the central nervous system, and the other two had bacteremia alone. Each patient recovered from his infection.

Persons with normal immunity who ingest *L. monocytogenes* in contaminated food typically have no manifestations or self-limited fever and diarrhea [10]. Those with persistent fever and positive stool cultures may benefit from antibiotic treatment [10]. Persons who have compromised defenses against *L. monocytogenes* include pregnant women; neonates; those with malignancy, diabetes mellitus, or chronic renal disease; those with acquired immunodeficiency syndrome; those who take glucocorticosteroid medications; and those > 50 years of age [10, 10–14]. Presenting symptoms of listeriosis in these persons include fever, myalgias, headache, confusion, loss of balance, and seizures. Most persons with compromised defenses who develop listeriosis require hospitalization, antibiotic therapy, and supportive care [10]. Serious complications include miscarriage and premature delivery; infection of neonates; and bacteremia, meningitis, and death [10–14].

Bacteremia without an evident focus is the most common manifestation of *L. monocytogenes* infections in compromised hosts [12]. In a 2004 review of 118 patients with malignancy and *L. monocytogenes* infections, the most common site of infection was also blood alone (53%) [13], consistent with the present case. Fifty-three percent of the 118 patients had leukemia (chronic lymphocytic leukemia, 21%; acute lymphoblastic leukemia and variants, 15%; acute myelogenous leukemia, 7%; chronic myelogenous leukemia, 6%; HCL, 3% [7, 8, 15]; and other leukemia, 2%) [13]. There were strong associations of *L. monocytogenes* infection with recent corticosteroid therapy (50%), recent chemotherapy (43%), or previous stem cell transplantation (17%). Mortality among patients with *L. monocytogenes* bacteremia alone was 18% [13].

*BRAF* p.V600E was detected at diagnosis in each of 48 patients with HCL reported by Tiacci et al. [16]. *BRAF* p.V600E was not detected in the present patient ten months after cladribine therapy. At that time, flow cytometry detected a hairy cell immunophenotype in only 0.9% of blood lymphocytes. The limit of detection of the assay used is 5% mutant alleles in a background of 95% wild-type alleles [17]. Thus, one cannot conclude that HCL in the present patient is not associated with *BRAF* p.V600E.

Features of the present case that distinguish it from variant HCL include lack of prominent nucleoli (Figure 1); positivity for CD11c, CD25, and CD103 (Table 1); and excellent response to cladribine [18]. Leukocyte counts were reported in 47 of 48 patients with HCL and *BRAF* p.V600E, among whom 12 (25.5%) had leukocyte counts at diagnosis > 11.0 × 10^9^/L (range 11.4–55.0 × 10^9^/L) [16]. Thus, we do not interpret leukocytes 15.6 × 10^9^/L (lymphocytes 10.5 × 10^9^/L) at diagnosis of the present patient as evidence that he has variant HCL.

Leukemic lymphocytes in the present patient were positive for CD5. Although CD5 positivity is not typical of HCL [19], this phenotype has been described in a small proportion of HCL cases [20]. Whether CD5 positivity represents a distinctive subtype of lymphoid malignancy is unknown [20]. Trisomy 12 occurred in three percent of blood lymphocytes in the present case. Trisomy 12 is not typical of HCL [3, 21] but has been described in a small proportion of patients [22, 23]. In such cases, the proportion of cells that display trisomy 12 is small [22, 23], like the present case. Except the appearance of dim CD23 positivity in leukemic lymphocytes in month 18, the leukemia cell immunophenotype in the present patient did not change.

Limitations of the present study include the possibility that we overlooked other reports of *L. monocytogenes* infections in patients with HCL in our manual literature review. Cases of other patients with HCL who developed *L. monocytogenes* infections may be unreported. Foodborne *L. monocytogenes* sometimes causes epidemic acute, febrile gastroenteritis that lasts two days [24]. Some patients with HCL and *L. monocytogenes* gastroenteritis may have been mistakenly diagnosed to have non-*Listeria* gastroenteritis.

## Figures and Tables

**Table 1:** Immunophenotypes of clonal blood B-lymphocytes in a man with hairy cell leukemia^1^.

Surface antigen	Month 1 (diagnosis of leukemia)	Month 16 (*Listeria* bacteremia)	Month 18 (initial cladribine therapy)	Month 24 (after initial cladribine therapy)	Month 28^2^
CD5	+	+	+	+	+
CD10	nd	−	nd	−	nd
CD11c	+	nd	+ Bright	+ Bright	+ Bright
CD19	+	+	+	+ Bright	nd
CD20	+ Bright	+ Bright	+ Bright	+ Bright	nd
CD22	+	+	+ Bright	+ Bright	nd
CD23	−	−	+ Dim	−	nd
CD25	+	nd	+	+	+
CD45	+	+	+	+	nd
CD103	+	nd	+	+	+
FMC-7	+	+	+	+	nd
HLA-DR	+	nd	+	+	nd
Lambda	+	+ Bright	+	+	nd

^1^Immunophenotypes were determined using flow cytometry; nd = not done; ^2^complete blood count: hemoglobin 120 g/L, erythrocytes 4.05 × 10^12^/L, mean corpuscular volume 97 fL, leukocytes 3.7 × 10^9^/L, neutrophils 2.5 × 10^9^/L, lymphocytes 0.9 × 10^9^/L, and platelets 88 × 10^9^/L. 0.9% of blood lymphocytes had a hairy cell leukemia immunophenotype.

**Table 2:** *Listeria monocytogenes* infections in 6 patients with hairy cell leukemia^1^.

Patient number	Age (y), sex	Previous leukemia management	Infection^2^	Comorbid condition(s)	Antibiotic(s)^3^	Reference
1	70, M	ns	Meningoencephalitis	ns	Ampicillin + trimethoprim/sulfamethoxazole	[5]
2	62, M	Splenectomy 11 y before	Meningitis	Asthma treated with prednisone 5 mg daily	Ampicillin + gentamycin	[6]
3	73, M	Splenectomy 6 y before	Meningitis	Acquired immunodeficiency syndrome; thrombocytopenia; Kaposi's sarcoma treated with vinblastine, electron beam	Ampicillin + gentamycin	[7]
4	53, M	Splenectomy 4 y before	Cutaneous lesions, bacteremia, cerebritis	Seropositive for hepatitis B	Ampicillin + gentamycin; corticosteroids	[8]
5	93, M	ns	Bacteremia	Antileukemia chemotherapy	ns	[9]
6	66, M	No therapy	Bacteremia	None	Levofloxacin	Present report

^1^Age at diagnosis of *L. monocytogenes* infection; ns = not stated; ^2^possible sources of infection were reported as soft cheese and meat/poultry cold cuts in patient number 2 and horses and silage in the present case; ^3^predominant antibiotic therapy during acute infection. In patient number 2, infection progressed on initial ciprofloxacin therapy. Before *L. monocytogenes* was identified in cultures, some patients received other antibiotics that were subsequently discontinued, as appropriate. Some patients received protracted antibiotic therapy after resolution of the acute phase of infection. Each of these six men survived his respective infection.
